# Retrospective analysis of ferric sulfate and sodium hypochlorite pulpotomy procedures in primary molars

**DOI:** 10.34172/joddd.2023.39312

**Published:** 2023-11-11

**Authors:** Hayri Akman, Koray Surme

**Affiliations:** Department of Pediatric Dentistry, Faculty of Dentistry, Alanya Alaaddin Keykubat University, Antalya, Turkey

**Keywords:** Primary molar, Pulpotomy, Radiographic success, Retrospective study

## Abstract

**Background.:**

Pulpotomy is a vital pulp treatment procedure frequently used in primary molars to preserve pulp vitality and function until tooth exfoliation. There is currently no pulp medicament with all the features of the ideal pulpotomy material. The present study compared the radiographic success of sodium hypochlorite with ferric sulfate (FS) when used for pulpotomy in primary molars.

**Methods.:**

A retrospective analysis was performed to evaluate the success rates of primary molars pulpotomized using sodium hypochlorite or FS according to radiographic findings. Healthy 4‒10-year-old children who had pulpotomy procedures on primary molars between 2018 and 2021 at the pediatric dental clinic and had a control radiograph at least 12 months later were enrolled in the study. The chi-squared test was used to determine the differences in success between these two materials.

**Results.:**

A total of 142 teeth, including 85 (59.9%) first primary molars and 57 (40.1%) second primary molars, in 98 healthy children were evaluated. The mean follow-up period of the teeth included in the study was 585.1±249.4 days. Radiographic success rates for NaOCl and FS groups were 73.8% and 71.0%, respectively, with no statistically significant difference (*P*>0.05). Internal root resorption (IRR) was the most common cause of radiographic failure in both groups.

**Conclusion.:**

Radiographic success rates of both materials were similar, and using these materials in primary molar pulpotomy procedures can be recommended in clinical practice.

## Introduction

 Pulpotomy is a vital pulp treatment procedure frequently utilized in the primary dentition to preserve pulp vitality and function when the pulp tissue is exposed due to trauma or excavation of a carious lesion. The damaged coronal pulp tissue is removed, and suitable conditions are created for healing the healthy radicular pulp tissue to achieve tooth function in the oral cavity until exfoliated.^1,2^ The primary objective of a successful pulpotomy is to prevent premature tooth loss and preserve the integrity of the dental arch, along with an asymptomatic tooth with preserved radicular pulp.^3^ The clinical method and indications for the pulpotomy procedure have remained unchanged over the years, whereas the implemented therapeutic materials have become increasingly varied and evolving over time.^4^ Based on their impact on the residual radicular pulp tissue, the current pulpotomy materials can be divided into three categories.^5^ These include agents that induce devitalization (e.g., formocresol and glutaraldehyde), maintain healing (e.g., ferric sulfate, bioactive cements, calcium hydroxide, sodium hypochlorite, lasers, etc.), or promote regeneration (e.g., bone morphogenic proteins).^6^

 Ferric sulfate (FS), a non-aldehyde chemical, has been used in primary tooth pulpotomy as a coagulative and hemostatic pulp medicament agent since 1988.^7^ FS is recognized as a non-toxic, effective, practical, and accessible alternative material for primary tooth pulpotomy. When in contact with blood proteins during the pulpotomy procedure, this material produces a ferric ion-protein complex without a blood clot, thereby minimizing the chances of inflammatory response.^8,9^ Application of FS solution for 15 seconds in amputation treatment is effective in the primary tooth pulp^8^: however, histological studies have shown that pulpotomy with FS induces a severe inflammatory response and also has a high incidence of premature loss due to internal‒external resorption and abscess formation.^10-12^

 Sodium hypochlorite (NaOCl) has been used as an endodontic irrigant in root canal treatment procedures for nearly 100 years and has shown effective antibacterial properties without irritating the pulp.^13^ Additionally, NaOCl has excellent tissue-dissolving properties and can be used successfully in controlling tissue amputation hemorrhage. However, only a few clinical trials have evaluated the efficacy of NaOCl as a medicament in pulpotomy procedures of primary teeth, and data support its application in the biochemical amputation of the dental pulp, which leads to the formation of a dentin‒pulp interface free of coagulum debris before the base material placement.^14,15^

 According to preliminary clinical investigations, NaOCl could be a viable option for pulpotomy medication for primary teeth.^16-18^ However, few studies have compared the success of FS, which has been used frequently in primary tooth pulpotomy for many years, with sodium hypochlorite in clinical applications. Therefore, this research evaluated the radiographic success of 5% NaOCl with 15.5% FS in pulpotomy procedures of vital primary molars.

## Methods

 This study was carried out with the approval of the Ethics Committee of Alanya Alaaddin Keykubat University Faculty of Medicine (2022/04-06). In this retrospective analysis, pulpotomy procedures were performed on the primary molars of healthy 4‒10-year-old children as part of their regularly scheduled dental treatment at the Pediatric Dental Clinic of the Faculty of Dentistry between 2018 and 2021. Data were acquired from completed treatment files from 2018 to 2021. Eligibility of subjects for this study was determined by screening through a dental hospital software program using the following criteria: Patients who visited the dental clinic between January 2018 and December 2021, had a diagnostic radiograph, and had a control radiograph at least 12 months after pulpotomy treatment. Radiographs with low or moderate quality, less than twelve months of follow-up sessions, or an incomplete clinical history were excluded.

 The researchers defined the pulpotomy material by checking the dental hospital software program database, examining the treatment notes, and determining whether FS or NaOCl medications were utilized. Examination of the medical records revealed that teeth with deep caries and no more than coronal reversible pulpitis were eligible for pulpotomy procedures.^19^ When examining diagnostic radiographs, the absence of radiographic evidence of pulp degeneration, such as internal or external resorption, inter-radicular, and/or periapical bone destruction, was checked. All the teeth included in the study were indicated by a single pediatric dentist, and every stage of treatment was applied by the same specialist in the pediatric dental practice with local anesthesia.

 The success rates of pulpotomy treatments were determined by evaluating the diagnostic radiographs taken before the FS or NaOCl pulpotomy treatment and the control radiographs at least 12 months later. All radiographs with good quality, good image quality, and radiographic device standardization were examined in detail by two pediatric dentists. In cases of inconsistency between radiographic evaluation results, a consensus was reached by reexamining. In the study, teeth on which pulpotomy treatment was performed and those that were radiographically asymptomatic for at least one year were considered successful. Radiographic failure criteria were determined based on the study of Shabzendedar et al,^18^ and teeth with internal or external root resorption and periapical or furcation radiolucency in the control film taken at least 12 months after pulpotomy treatment were considered unsuccessful.

 The routine primary tooth pulpotomy procedure in the clinical application was followed. After the local anesthesia procedure, the indicated teeth were isolated by cotton rolls in all patients. The carious lesion was eliminated, and the pulp chamber was opened utilizing a round diamond bur in a high-speed handpiece with water cooling. Coronal pulp amputation was performed using a low-speed sterile round bur, followed by irrigation with saline solution to remove debris. Bleeding control was achieved with sterile saline-blotted pellets placed on the pulpal stumps with minimal pressure.^20^ After hemostasis in the NaOCl group, a cotton pellet soaked in 5% NaOCl was applied in the pulp chamber for 30 seconds. After hemostasis in the FS group, a solution of 15.5% FS was administered to the pulpal stumps for 15 seconds using the manufacturer-supplied dental infuser. The cavity was then gently washed with physiological saline and dried. Before restoring the teeth in all groups, a coating of resin-bonded zinc oxide eugenol (Kalzinol; Dentsply, Konstanz, Germany) cement was applied over amputated pulp stumps. Also, the agents used while pulpotomy treatments were performed on the teeth were recorded as treatment notes in the dental hospital software program. Glass-ionomer cement (Fuji IX, GC Tokyo, Japan) was placed over the ZOE cement, and restorations were completed with composite resin (Gradia, GC Tokyo, Japan).

###  Statistical analysis

 Chi-square test was used to compare the radiographic success of pulpotomy with NaOCl and FS materials. All statistical analyzes were performed using SPSS 21 (IBM; Armonk, NY, USA), and the statistical significance level was set at *P* < 0.05.

## Results

 A total of 142 teeth, including 85 (59.9%) first primary molars and 57 (40.1%) second primary molars, in 98 healthy children were treated in this study. The children consisted of 53 (54.1%) males and 45 (45.9%) females, with a mean age of 6.38 ± 1.41 years at the beginning of treatment. NaOCl was used in 80 (56.3%) and FS in 62 (43.7%) teeth followed in the study. The follow-up periods of the teeth in the study ranged from 367 to 1258 days, and the mean follow-up period was 585.1 ± 249.4 days. The demographic characteristics and details of the patients according to the pulpotomy material are shown in Table 1.

**Table 1:** Demographics and details of patients according to the amputation material

**Characteristics**	**NaOCl** **No. (%) or mean (±SD)**	**Ferric sulfate ** **No. (%) or mean (±SD)**	**Total ** **No. (%) or mean (±SD)**
Age (y)	6.23 ( ± 1.35)	6.61 ( ± 1.49)	6.38 ( ± 1.41)
Gender			
Male	30 (56.6%)	23 (43.4%)	53 (100%)
Female	29 (64.4%)	16 (35.6%)	45 (100%)
Follow-up period (days)	580.9 ( ± 244.7)	590.4 ( ± 257.3)	585.1 ( ± 249.4)
Jaws			
Maxillary	21 (42.9%)	28 (57.1%)	49 (100%)
Mandibular	59 (63.4%)	34 (36.6%)	93 (100%)
Type of tooth			
First primary molar	45 (52.9%)	40 (47.1%)	85 (100%)
Second primary molar	35 (61.4%)	22 (38.6%)	57 (100%)
Total	80 (56,3%)	62 (43,7%)	142 (100%)

 The radiographic success rates for the NaOCl and FS groups were 73.3% and 67.5% in the first primary molars, 74.3% and 77.3% in the second primary molars, and 73.8% and 71.0% in all teeth (Table 2). First primary molars, second primary molars, and all teeth exhibited no statistically significant differences in success between NaOCl and FS (*P* > 0.05). Table 3 shows the pathological radiographic findings in the pulpotomy treatment of primary molars according to materials. Internal root resorption (IRR) was the most common cause of radiographic failure in NaOCl (61.9%, n = 13/21) and FS (66.7%, n = 12/18).

**Table 2:** Radiographic pulpotomy success status of materials

**Type of tooth**	**Agent**			***P* value**^a^
	**NaOCl** **No. (%) **	**Ferric sulfate ** **No. (%) **	**Total ** **No. (%) **	
First primary molar				
Success	33 (73.3%)	27 (67.5%)	60 (70.6%)	0.556
Failure	12 (26.7%)	13 (32.5%)	25 (29.4%)	
Second primary molar				
Success	26 (74.3%)	17 (77.3%)	43 (75.4%)	0.799
Failure	9 (25.7%)	5 (22.7%)	14 (24.6%)	
Total				
Success	59 (73.8%)	44 (71.0%)	103 (72.5%)	0.713
Failure	21 (26.3%)	18 (29.0%)	39 (27.5%)	

^a^Chi-squared test was used for the statistical analysis.

**Table 3:** Radiographic findings for the pulpal failure of pulpotomy materials

**Radiographic finding**	**Agent**		
	**NaOCl** **No. (%)**	**Ferric sulfate ** **No. (%)**	**Total ** **No. (%)**
Internal root resorption	13 (61.9%)	12 (66.7%)	25 (64.1 %)
External root resorption	2 (9.5%)	1 (5.5%)	3 (7.7 %)
Periapical or furcation radiolucency	6 (28.6%)	5 (27.8%)	11 (28.2%)
Total	21 (100%)	18 (100%)	39 (100%)

## Discussion

 This retrospective analysis compared the radiographic success rates of 15.5% FS and 5% NaOCl as pulpotomy medicaments in primary molars in cases followed for at least 12 months. The pulpal responses of primary teeth differ significantly from those of permanent teeth, as the pulp of primary teeth becomes inflamed and degenerates more rapidly and reacts less favorably. The high level of cellularity and vascularity of the primary tooth pulp contributes to its high repair potential. Therefore, a pulpotomy is a suitable therapeutic procedure for managing infection of the coronal pulp tissue in teeth because of its exceptionally high positive outcomes.^21^ The preservation of healthy radicular pulp tissue offers nutritional support for teeth, contributes to the formation of a dentin bridge, and preserves the primary teeth’s integrity.^22^

 The optimal pulpotomy material must inhibit bacterial formation, be biocompatible with the pulp and its surrounding tissues, support healing and viability of the radicular pulp, and not interfere with the physiological root resorption process.^3,4^ Although many materials and techniques are used in pulpotomy procedures in primary teeth, there are currently no pulp coating agents with all the features of the ideal pulpotomy material. In addition, there is no consensus regarding the optimal therapeutic pulpotomy agent.^23^ These findings have encouraged researchers to search for an alternative pulpotomy medicament.^24^ Therefore, the current study aimed to compare the radiographic success rates of NaOCl and FS as pulpotomy agents.

 Control of pulpal bleeding during amputation treatment is one of the most important treatment steps. If bleeding is not controlled, the blood clot formed on the pulp surface will form a barrier between the pulp tissue and the coating material, which may result in a chronic inflammatory response.^25^ To prevent clot formation, a disadvantage of traditional methods, using hemostatic agents during hemorrhage control has gained popularity. FS provides hemostasis by chemical reaction with blood and has become a hemostatic agent widely used in primary tooth pulpotomy procedures.^26,27^

 In the literature, variable results have been reported regarding the success rate of FS in primary tooth pulpotomy procedures. A systematic review reveals that the clinical success rates after pulpotomy with FS range from 78% to 100% (on average 91.6%), while the radiographic success rates range from 42% to 97% (on average 73.5%).^28^ Similarly, the current study results on radiographic success rates of FS pulpotomy procedures are within the bounds of these previous research findings. In the present study, the radiographic success rate for FS pulpotomy procedures was 70%, which fell within the range reported in the literature.^3^

 After coronal pulp amputation, bacteria may persist in the pulp stumps. In vital pulp therapy, the pulp is more likely to exhibit an inflammatory response in the event of bacterial contamination, according to studies.^18^ Even in cases where bacteria already exist, treatment success is more likely if an antibacterial agent is applied.^29^ The radiographic failures encountered in the present study might have resulted from this material’s lack of healing-promoting properties and masking the pathology of the underlying pulp tissue with its hemostatic effect.^30,31^

 In this study, the radiographic success rate of 5% NaOCl pulpotomy with at least 12 months of follow-up was 75%, consistent with earlier findings. In 2006, Vargas et al^26^ used NaOCl as a pulpotomy medicament for the first time in the primary dentition. At a 12-month follow-up, they noted that 5% NaOCl had a 79% radiographic success rate. Vostatek et al^32^ reported a similar radiographic success rate (82%) over 21 months using 5% NaOCl as the medicament in primary molar pulpotomy procedures. Another randomized study by Al-Mutairi and Bawazir^16^ evaluated the 5% NaOCl as the medicament in primary molar pulpotomy procedures and reported a radiographic success rate of 86.5% after 12 months. Ruby et al^17^ evaluated the 3% NaOCl as a medicament in primary molar pulpotomy and reported an 80% radiographic success rate at 12 months.

 Li et al^20^ used 5% NaOCl in pulpotomy procedures of primary dentition and reported that radiographic success rates for NaOCl pulpotomy procedures were similar to those found for FS pulpotomy procedures in previous studies with comparable follow-up intervals. Farsi et al^33^ evaluated the success of FS and sodium hypochlorite in primary tooth pulpotomy procedures for 18 months and reported no significant differences in radiographic success rates between these groups. In retrospective and prospective clinical studies that used different amputation agents, there were no differences between the radiographic success rates of primary tooth amputations using FS and NaOCl at a 12-month follow-up.^32,34^ The current study’s findings are consistent with previous research. However, in both groups, the mandibular first primary molar was the tooth that underwent treatment most frequently in this analysis. This observation might be explained by the fact that mandibular molars are more likely than maxillary molars to develop caries.^35^

 Agents frequently used in primary tooth pulpotomy procedures, such as MTA, a biocompatible dentin bridge-inducing material, and formocresol, which fixes and mummifies the tissue completely, have shown successful results in previous studies.^27^ However, MTA has a high cost and is more difficult to handle, and formocresol has some adverse effects, such as potential carcinogenicity, mutagenicity, and cytotoxicity.^20^ FS is a low-cost and easily accessible alternative coagulative and hemostatic medicament for primary tooth pulpotomy.^36^ NaOCl is biocompatible with pulp tissue and has high efficiency in tissue bleeding control; therefore, it can be used in primary tooth pulpotomy procedures.^37^ So, the advantages of using FS and NaOCl in primary tooth pulpotomy procedures are that they are biocompatible with pulp tissue compared to formocresol and are more cost-effective than MTA.^33,34^

 IRR has been reported in previous studies as the most frequent unfavorable outcome when either FS or NaOCl is used as a pulpotomy agent in primary teeth. However, chronic inflammation in the radicular pulp tissue may cause IRR due to diagnostic errors made during the evaluation of the pulp status or a technical failure during the treatment procedure.^6^ In addition, the direct interaction of eugenol with the vital dental pulp can cause mild to severe irritation and hence internal resorption if the zinc oxide eugenol cement is used as a sub-base following pulpotomy.^5,38^ In this study, internal resorption was the most common radiologic failure finding in both groups, consistent with the available literature.

 This study has some limitations due to its retrospective design based on existing case records, in which there may be a recording inaccuracy. The lack of equal follow-up periods and different treatment options due to the study design may have caused the lack of standardization. The pulpotomy procedures in previous studies were performed by multiple operators.^32,39^ In order not to affect the results due to the inconsistency of the techniques and the initial diagnosis in case selection, all the teeth included in the study were marked by a single pediatric dentist, and each stage of their treatment was performed by the same specialist under local anesthesia in the pediatric dentistry clinic.

## Conclusion

 In conclusion, radiographic success rates for NaOCl pulpotomy procedures (73.8%) in this study were similar and comparable to those with FS (71.0%) at a minimum follow-up period of 12 months. Considering its availability and affordability, NaOCl could be a suitable alternative to FS for primary molar pulpotomy. However, additional high-quality prospective clinical trials with longer follow-ups are required to improve the knowledge of various therapeutic pulpotomy materials and for more definite clinical practice recommendations.

## Competing Interests

 The authors declare that they have no competing interests.

## Ethical Approval

 This study was carried out with the approval of the Ethics Committee of Alanya Alaaddin Keykubat University, Faculty of Medicine (2022/04-06). Written and verbal consent was obtained from the parents of all patients under 18 years of age included in the study as a routine procedure during examination, diagnosis, and treatment.
